# CPX-351 and the Frontier of Nanoparticle-Based Therapeutics in Acute Myeloid Leukemia

**DOI:** 10.3390/ijms262311628

**Published:** 2025-11-30

**Authors:** Ioannis Konstantinidis, Sophia Tsokkou, Antonios Keramas, Eleni Gavriilaki, Georgios Delis, Theodora Papamitsou

**Affiliations:** 1 Department of Medicine, Faculty of Health Sciences, Aristotle University of Thessaloniki, 54124 Thessaloniki, Greece; stsokkou@auth.gr (S.T.); antonios@auth.gr (A.K.); 2 Haematology Unit—Haemophilia Centre of Northern Greece, 2nd Propedeutic Department of Internal Medicine, Hippokration General Hospital of Thessaloniki, Aristotle University of Thessaloniki, 54124 Thessaloniki, Greece; gavriiel@auth.gr; 3 Laboratory of Pharmacology, School of Veterinary Medicine, Faculty of Health Sciences, Aristotle University of Thessaloniki, 54124 Thessaloniki, Greece; delis@vet.auth.gr; 4 Laboratory of Histology-Embryology, Department of Medicine, Faculty of Health Sciences, Aristotle University of Thessaloniki, 54124 Thessaloniki, Greece

**Keywords:** CPX-351, acute myeloid leukemia (AML), liposomal drug delivery, nanoparticle therapeutics, cytarabine, daunorubicin, therapy-related AML, AML-MRC, MRD negativity, translational oncology

## Abstract

Acute myeloid leukemia (AML) continues to carry a dismal prognosis in older adults and those with secondary or high-risk disease, where conventional 7 + 3 chemotherapy has long delivered complete remission rates below 40% and median overall survival often under 6 months. CPX-351 (Vyxeos), a liposomal co-encapsulation of cytarabine and daunorubicin at a fixed synergistic 5:1 molar ratio, was designed to overcome the pharmacokinetic mismatch that undermines the traditional regimen. This review critically examines the preclinical rationale and clinical evidence for CPX-351, with particular attention to whether its nanoparticle platform truly represents a breakthrough or merely an incremental refinement of decades-old cytotoxics. Across phase I–III trials and real-world cohorts, CPX-351 consistently outperformed standard 7 + 3 in its approved populations of newly diagnosed therapy-related AML (t-AML) and AML with myelodysplasia-related changes (AML-MRC) in patients aged 60–75 years. In the pivotal phase III study (n = 309), CPX-351 improved median overall survival from 5.95 to 9.56 months (HR 0.69, 95% CI 0.52–0.90; *p* = 0.005) and raised the complete remission rate from 33.3% to 47.7%, while facilitating allogeneic transplantation in 34% as opposed to 25% of patients. A five-year follow-up sustained the separation in survival curves, and post-hoc analyses of responders showed median overall survival exceeding 25 months with CPX-351 versus approximately 10 months with 7 + 3 (HR 0.49). Real-world series have reported composite remission rates of 53–60%, measurable residual disease negativity in up to 65% of responders, and median overall survival of 12–20 months, depending on transplant utilization. Despite these gains, the absolute survival benefit remains modest, prolonged cytopenias are universal, and outcomes in TP53-mutated or younger adverse-risk patients are still poor, raising legitimate questions about cost-effectiveness and generalizability. Nonetheless, CPX-351 stands as the first clinically validated example of ratiometric nanomedicine in oncology, proving that reformulating established drugs can yield meaningful progress where novel agents have often failed.

## 1. Introduction

**Figure 1:**
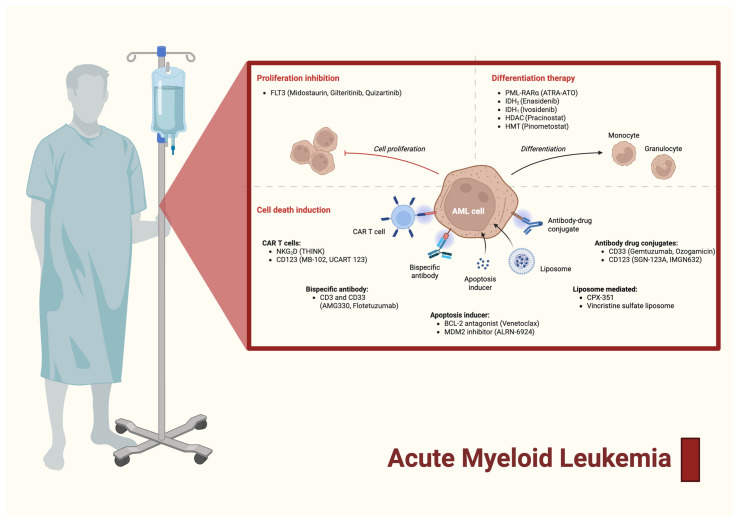
Mechanistic overview of therapeutic strategies in acute myeloid leukemia (AML) [1,2,3,4,5,6]. This illustration categorizes current AML therapies by their mechanistic targets: proliferation inhibition (*FLT3* inhibitors), differentiation therapy (ATRA, IDH inhibitors), cell death induction (CAR T cells, apoptosis inducers, antibody–drug conjugates), and immune modulation (checkpoint inhibitors, liposomal agents like CPX-351). The schematic highlights the multifaceted approach to AML treatment, integrating targeted, immunologic, and cytotoxic modalities to disrupt leukemic cell survival and promote remission.

Acute myeloid leukemia (AML) is a malignant clonal disorder characterized by the proliferation and accumulation of abnormal immature myeloid precursor cells (blasts) in the bone marrow, peripheral blood, or other tissues, with a block in normal myeloid differentiation, resulting in ineffective hematopoiesis and life-threatening cytopenias [1,2]. The pathogenesis of AML is driven by a complex interplay of complex genetic and epigenetic alterations. Chromosomal abnormalities (translocations, inversions, deletions) and somatic mutations in genes governing proliferation, apoptosis, and differentiation (such as *FLT3*, *NPM1*, *TP53*, and *DNMT3A*) facilitate the development of neoplastic clonal myeloid stem cells. Molecular insights have enhanced diagnostic criteria and guided risk stratification and therapeutic decision-making (Figure 1) [3,4].

Chemotherapy remains the primary approach for managing and treating AML, with regimens customized according to patient attributes such as fitness, age, cytogenetic-molecular risk, and specific disease subtypes [5,6]. For nearly four decades, the standard induction therapy for AML has been a combination of cytarabine and an anthracycline, such as daunorubicin or idarubicin, known as “7 + 3”, which achieves complete remission (CR) rates of 60 up to 80% in younger and fit patients [3,5,6]. This therapeutic stagnation was not due to a lack of effective agents, but rather a fundamental failure to optimize their combined pharmacokinetics. The conventional 7 + 3 regimen represents a clinical compromise, dictated by the disparate half-lives and systemic toxicities of its free-drug components [7]. Additionally, the chemotherapy-based consolidation involves intermediate or high-dose cytarabine, such as 1000 up to 1500 mg/m^2^ IV every 12 h for 3 days, repeated for 3 to 4 cycles, with or without allogeneic hematopoietic stem cell transplantation depending on risk stratification [3,4]. Nonetheless, results continue to be inadequate in older adults and individuals with unfavorable risk characteristics, necessitating the development of innovative therapeutic approaches.

Nanotechnology has emerged as a transformative approach in cancer therapeutics, offering unique advantages in drug delivery, cellular targeting, and immunomodulation. Nanoparticles, including liposomes, polymeric carriers, dendrimers, and micelles, enable targeted delivery and controlled release of chemotherapeutics. This field is being actively investigated as a strategy to improve both the diagnosis and treatment of AML using nanoparticles, including liposomes, polymersomes, micelles, dendrimers, and inorganic nanomaterials. These nanoparticles are engineered for drug delivery enhancement, and they increase specificity for leukemic cells and reduce off-target toxicity compared to conventional chemotherapy [8,9,10,11,12].

When it comes to patients not meeting the criteria for intensive chemotherapy, low-intensity regimens are standard with a combination of a hypomethylating agent (azacitidine or decitabine) with venetoclax, yielding response rates exceeding 60% and prolonging survival [13,14]. Recent developments involve the combination of venetoclax with new agents such as ICT01, an immunotherapy targeting BTN3A, which has demonstrated favorable early-phase outcomes in augmenting anti-leukemic efficacy [15,16,17].

CPX-351, commercially known as Vyxeos liposomal, a liposomal formulation of cytarabine and daunorubicin, has shown superior outcomes in older patients (60–75 years old) with therapy-related AML (t-AML) or AML with myelodysplasia-related changes (AML-MRC), improving overall survival (OS) in comparison to the traditional 7 + 3 regimen [7]. The objective of the present review is to critically examine CPX-351 as a clinically validated liposomal formulation in the treatment of AML, highlighting its design principles, pharmacologic advantages, and clinical impact in high-risk subtypes. By positioning CPX-351 within the broader landscape of nanoparticle-based therapeutics, we explore its role as a translational model for future drug delivery platforms.

A literature review was performed to assess the current state and therapeutic potential of new nanoparticle-based strategies in acute AML. The review encompassed original research articles, systematic reviews, and meta-analyses published in English up to 2025. A comprehensive search was conducted across multiple biomedical databases, including PubMed, MEDLINE, Scopus, Cochrane Library, and Embase, using the following query strings: “(CPX-351 OR Daunorubicin OR Cytarabine) AND (AML OR Acute Myeloid Leukemia) AND (Nanoparticle OR Nanomedicine OR Liposomal OR Drug delivery OR Targeted Therapy OR Immunotherapy)”.

Preclinical and clinical studies evaluating nanoparticle-based delivery of cytotoxic agents, gene therapies, or immunomodulators in AML, as well as reviews discussing mechanistic insights or translational relevance, were included. Excluded studies include those lacking AML-specific data, non-nanoparticle platforms, or insufficient methodological detail. Key studies’ reference lists were meticulously examined to locate further pertinent studies. After the exclusion of articles based on the above criteria by both the automated tools and the researchers, a final set of articles was retrieved. To ensure accuracy and objectivity, two independent reviewers (I.K. and S.T.) initially screened the titles and abstracts in a double-blinded process. For studies that passed this initial screening, the full texts were obtained and further evaluated to determine their final eligibility. Any discrepancies during the screening process were resolved by a third reviewer (T.P.). Key data were independently extracted from all included studies by two reviewers (I.K. and S.T.) and concentrated on nanoparticle design, mechanisms of action, therapeutic outcomes, toxicity profiles, and clinical trial status. The review emphasized new publications from the past five years while incorporating older, highly referenced works for contextual and basic understanding. Discrepancies in study results were reconciled through consensus among reviewers. To contextualize the therapeutic impact of CPX-351 across diverse clinical settings, a structured comparison of key studies evaluating its efficacy, safety, and translational relevance was created. Table 1 summarizes the findings across phase I–III trials, post hoc analyses, regulatory reviews, and real-world evidence (RWE) cohorts, including recent genomic and MRD-integrated datasets. The table highlights variations in study design, patient populations (therapy-related AML, AML-MRC, *TP53*-mutated, secondary-type mutations), dosing schedules, response rates, survival outcomes, and transplant feasibility. Notably, emerging data from long-term RWE cohorts reinforce the prognostic value of MRD negativity and support CPX-351’s role in achieving deeper remissions and sustained survival, particularly in high-risk AML subsets.

## 2. The Paradigm Shift from 7 + 3 to Ratiometric Nanomedicine

CPX-351 exemplifies the potential of nanomedicine to re-engineer established cytotoxic agents to enhance clinical outcomes in AML. In contrast to the traditional “7 + 3” regimen, which involves the administration of cytarabine and daunorubicin as separate entities with differing pharmacokinetic characteristics, CPX-351 encapsulates both medicines within a single liposome at a synergistic 5:1 molar ratio of cytarabine to daunorubicin [29,30,31]. The fixed ratio was precisely established by comprehensive preclinical research, showcasing optimum synergistic cytotoxicity in AML cell lines and xenograft models [9,32,33]. The liposomal formulation guarantees the preservation of this synergistic ratio during cellular uptake by leukemic blasts, providing a significant advantage over the non-simultaneous and fluctuating intracellular concentrations attained with free-drug combinations [9,32,34].

The design principles of CPX-351 provide several significant pharmacologic benefits. Current evidence demonstrates that CPX-351 uptake by leukemia cells is primarily mediated by the scavenger receptor class B type 1 (SR-BI), which recognizes apolipoproteins (A-I and A-II) adsorbed onto the liposomal surface as part of the protein corona formed in serum. This interaction facilitates a nonendocytic pathway, allowing selective transfer of liposomal contents into leukemia cells, with SR-BI expression levels correlating directly with CPX-351 uptake and cytotoxicity in multiple leukemia cell lines [35,36]. Inhibition of SR-BI, either by siRNA knockdown or pharmacologic blockade (BLT-1), significantly reduces CPX-351 uptake, confirming the receptor’s central role [35,36]. Additional uptake mechanisms include macropinocytosis and clathrin-mediated endocytosis, but these are less dominant compared to SR-BI-mediated transfer. Notably, high-dose BLT-1 paradoxically increases CPX-351 uptake via enhanced macropinocytosis, suggesting that modulation of SR-BI activity can alter intracellular trafficking pathways and potentially augment drug delivery [35,36]. These mechanisms influence efficacy by enabling preferential and sustained delivery of cytarabine and daunorubicin to malignant myeloblasts, maintaining the synergistic 5:1 molar ratio, and bypassing common resistance pathways such as P-glycoprotein-mediated efflux [32,37]. SR-BI expression may serve as a predictive biomarker for CPX-351 responsiveness, and variability in receptor expression or function could contribute to resistance. Furthermore, CPX-351 uptake is independent of the human equilibrative nucleoside transporter (hENT), which is often downregulated in cytarabine-resistant AML, providing an additional advantage in refractory disease [38].

Encapsulation in liposomes generally extends the circulation half-life of both medicines, facilitating greater accumulation in the bone marrow and spleen through potential mechanisms, which include enhanced permeability and retention (EPR) effects [32,39]. This preferential distribution improves drug delivery to the leukemic microenvironment while reducing systemic exposure [32,34]. This decrease in systemic exposure to free cytotoxic drugs results in a more advantageous toxicity profile for healthy tissues, alleviating prevalent dose-limiting toxicities such as cardiotoxicity from daunorubicin and neurological problems from cytarabine. Moreover, the liposomal delivery system may facilitate the enhanced internalization of medicines by leukemic cells, thus circumventing specific efflux pump-mediated resistance mechanisms that can diminish the efficacy of traditional chemotherapy [39]. Consequently, CPX-351 exemplifies a clinically verified model that illustrates the therapeutic potential of strategically engineered nanoparticle-based medication delivery AML.

## 3. Mechanism of Action of CPX-351 in AML

Cytarabine functions as a nucleoside analogue that inhibits DNA synthesis during the S-phase, whereas daunorubicin, an anthracycline, integrates into DNA and inhibits topoisomerase II, resulting in DNA strand breaks and apoptosis [1,40].

**Figure 2:**
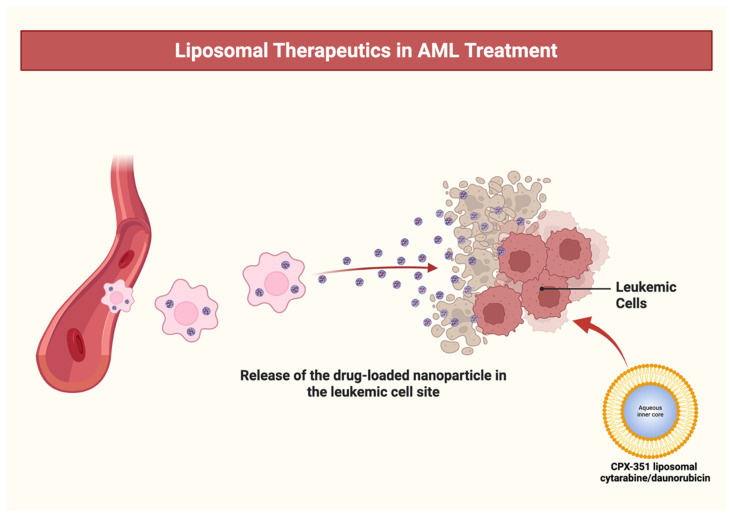
Liposomal therapeutics in AML. Treatment illustration of CPX-351 (liposomal cytarabine/daunorubicin) targeting leukemic cells via nanoparticle delivery. The liposomal formulation enables controlled release of cytotoxic agents directly at the leukemic cell.

CPX-351 aimed at enhancing the synergistic cytotoxicity of these drugs against AML blasts. In addition to that, CPX-351 activates DNA damage response pathways, including checkpoint kinase 1 (CHK1), and induces cell cycle arrest at the S and G2/M phases, further promoting apoptosis in AML cells. The enhanced pharmacodynamics and bone marrow tropism of CPX-351 translate into improved remission rates and OS in high-risk and secondary AML (s-AML) populations, as demonstrated in randomized clinical trials [21,41]. The liposomal delivery strategy extends the plasma half-life of both medications and selectively targets the bone marrow, leading to increased absorption by leukemic cells relative to normal hematopoietic cells (Figure 2). In vitro, it has shown enhanced selective cytotoxicity for AML compared to normal progenitors [41,42].

The fixed 5:1 molar ratio of CPX-351 preserves optimal medication synergy, which is challenging to attain with conventional administration owing to differences in pharmacokinetics. Liposomal encapsulation diminishes off-target toxicity and may lead to a reduced occurrence of cardiotoxicity and infectious complications relative to conventional regimens [40,42,43].

## 4. Comparative Dosing and Administration of Cytarabine: Conventional Regimens vs. CPX-351 in AML

The dose and route of administration of cytarabine differ significantly between conventional cytarabine regimens and CPX-351 in AML treatment. Firstly, conventional cytarabine (“7 + 3” regimen) is usually administered at 100 mg/m^2^/day by continuous intravenous (IV) infusion for 7 days consecutively during induction therapy [44]. It can also be given as an IV bolus or subcutaneously (SC) and is sometimes administered intrathecally (IT) for CNS prophylaxis or treatment, but for AML treatment, the standard of practice is continuous IV infusion [44,45].

For induction, CPX-351 is administered as an intravenous infusion over 90 min at a dose of 100 units/m^2^ (equivalent to cytarabine 100 mg/m^2^ and daunorubicin 44 mg/m^2^) on days 1, 3, and 5. If a second induction is required, the same dose is given on days 1 and 3. For consolidation, the dose is 65 units/m^2^ (cytarabine 65 mg/m^2^ and daunorubicin 29 mg/m^2^) on days 1 and 3, also as a 90 min IV infusion (Table 2) [21,41,45,46].

## 5. Commonly Studied Groups in CPX-351 Versus Conventional Cytarabine for AML

Recent therapeutic advances have significantly reshaped the treatment landscape for older adults and patients with adverse-risk AML. Through a literature search, it was observed that the most extensively studied patient populations (60 years of age and greater) with AML in nanoparticle-based therapies research are older adults, patients with relapsed or refractory AML, and those with high-risk or poor-prognosis disease. The greater inclusion of this particular group is attributed to the higher interest and prioritization due to a great unmet clinical need and to poor tolerance of standard chemotherapy, higher rates of chemoresistance, and limited therapeutic options available [9,10,31,47].

## 6. FDA-Drug Approval and the Pivotal Trial

Vyxeos (CPX-351) received FDA approval in 2017 for the treatment of adults with newly diagnosed t-AML or AML-MRC [22]. This approval was based on the pivotal study 301, a randomized, multicenter trial comparing Vyxeos to standard “7 + 3” chemotherapy in patients aged 60–75. Vyxeos significantly improved median OS (9.6 vs. 5.9 months; HR = 0.69, *p* = 0.005) and achieved higher CR rates (38% vs. 26%), with consistent benefits across subgroups including t-AML and AML-MRC [21,41]. The safety profile was generally comparable to “7 + 3,” though Vyxeos was associated with more prolonged neutropenia and thrombocytopenia, leading to increased bleeding risks and a need for careful monitoring. Pharmacokinetic studies demonstrated prolonged plasma half-lives and enhanced bone marrow retention of both agents, supporting the mechanistic rationale for improved efficacy. Regulatory considerations emphasized the non-interchangeability of Vyxeos with conventional formulations and included warnings for copper-related toxicity and cardiotoxicity. These results, alongside consistent efficacy in high-risk AML subtypes, established Vyxeos as the first FDA-approved therapy specifically indicated for t-AML and AML-MRC [22].

## 7. CPX-351: Liposomal Innovation Driving Remission and Survival in High-Risk and Secondary AML Across Trials and Real-World Cohorts

### 7.1. Liposomal Mechanism and Therapeutic Rationale

Liposomes are among the most therapeutically effective nanocarriers in AML therapy, as demonstrated by CPX-351 (liposomal cytarabine/daunorubicin), optimizes drug transport, mitigates cardiotoxicity, and improves bone marrow targeting. Liposomes can encapsulate both hydrophilic and hydrophobic pharmaceuticals, and their surfaces are frequently functionalized with ligands or antibodies (anti-CD33, transferrin) to enhance selectivity for AML cells and reduce off-target damage. In a phase III clinical trial conducted on patients with s-AML, CPX-351 showed promising results, enhancing the induction response by around 40%. Recent advancements encompass stimuli-responsive liposomes and immunoliposomes, facilitating regulated medication release and targeted action against leukemic blasts and stem cells [20,29,30].

### 7.2. Early-Phase Trials and Dose Optimization

The first ever study human-phase trial evaluating CPX-351 was conducted by Feldman et al. [18]. The study examined the liposomal formulation of cytarabine and daunorubicin in a fixed 5:1 molar ratio, in 48 patients with relapsed or refractory AML, ALL, or high-risk MDS. CPX-351 was administered on days 1, 3, and 5 via 90 min infusion, with dose escalation from 3 to 134 units/m^2^. The maximum tolerated dose was determined to be 101 units/m^2^, with dose-limiting toxicities including congestive heart failure, hypertensive crisis, and prolonged cytopenias. The overall response rate in AML was 23% (10/43), including patients previously treated with cytarabine and anthracyclines. Notably, responses were observed even at lower doses (≥32 units/m^2^), and the 5:1 molar ratio was maintained in plasma for over 24 h. Pharmacokinetic analysis revealed prolonged half-lives for both cytarabine (mean ~31 h) and daunorubicin (~22 h), with most of the drug remaining encapsulated. CPX-351 demonstrated a favorable safety profile and promising activity in heavily pretreated patients, supporting its advancement to phase II trials [18].

In the study of Cortes et al. [20], which was a phase II multicenter randomized trial, CPX-351 was investigated in adults with first relapse AML. Patients were stratified by the European Prognostic Index (EPI) and randomized 2:1 to receive CPX-351 or the investigator’s choice of intensive salvage therapy. While CPX-351 did not significantly improve 1-year survival in the overall population, it demonstrated notable benefits in the poor-risk subgroup, which comprised 68% of participants. In this group, CPX-351 yielded higher response rates (CR + CRi: 39.3% vs. 27.6%), improved event-free survival (EFS) (HR = 0.63, *p* = 0.08), and significantly better OS (HR = 0.55, *p* = 0.02). CPX-351 also showed enhanced early marrow clearance and reduced 60-day mortality. Safety profiles were comparable, though CPX-351 was associated with delayed hematologic recovery and increased infection-related events without a rise in infection-related deaths. Importantly, patients with prior hematopoietic stem cell transplant had poorer outcomes with CPX-351, suggesting the need for caution in this subgroup. These findings support further investigation of CPX-351, particularly in poor-risk AML populations without prior transplant [20].

Moving on, the phase II trial conducted by Issa et al. assessed CPX-351 in adults with newly diagnosed AML deemed at high risk for induction mortality due to factors including t-AML, s-AML, AML with MDS-related alterations, adverse cytogenetics, poor performance status (ECOG 2–3), renal impairment, or advanced age (≥70 years). Patients were randomized to receive CPX-351 at dosages of 50, 75, or 100 units/m^2^ on days 1, 3, and 5. The 75 units/m^2^ dosage exhibited the most favorable balance of efficacy and safety, with a composite remission rate (CR + CRi) of 38% and a median OS of 8.6 months, in contrast to 19% and 4.3 months at 50 units/m^2^, and 44% and 6.2 months at 100 units/m^2^, respectively. Patients with *TP53* mutations exhibited markedly poorer outcomes (median OS 2.6 months), whereas individuals with diploid cytogenetics demonstrated enhanced survival (median OS 14.5 months). The research indicated that previous exposure to hypomethylating drugs correlated with diminished reaction rates. CPX-351 was predominantly well tolerated, with febrile neutropenia, pneumonia, and sepsis identified as the most prevalent grade 3–4 adverse events. The trial endorses the administration of CPX-351, specifically at a dosage of 75 units/m^2^, in high-risk AML cohorts often omitted from intense chemotherapy studies, including those aged over 75 years [23].

In the multicenter, randomized Phase II trial of Lancet et al. [19], CPX-351 was evaluated against conventional 7 + 3 chemotherapy in older adults (aged 60–75) with newly diagnosed AML. Patients were stratified by risk, including sAML, adverse cytogenetics, and age ≥70 years. CPX-351 demonstrated a higher composite remission rate (CR + CRi: 66.7% vs. 51.2%; *p* = 0.07), meeting predefined criteria for success. Although differences in EFS and OS were not statistically significant in the overall cohort, CPX-351 conferred marked benefit in the sAML subgroup, with improved OS (12.1 vs. 6.19 months; HR = 0.46; *p* = 0.01) and EFS (4.5 vs. 1.3 months; HR = 0.59; *p* = 0.08). CPX-351 was associated with slower hematologic recovery but lower 60-day mortality (4.7% vs. 14.6%) and no increase in infection-related deaths. These findings support the ratiometric dosing hypothesis and highlight CPX-351’s therapeutic potential in high-risk AML, particularly sAML, providing the rationale for subsequent Phase III investigation.

### 7.3. Pivotal Phase III Trial—The Turning Point

From that point on, the pivotal Phase III trial of Lancet et al. [41] marked a turning point in the treatment of older adults with high-risk AML. This randomized, open-label study enrolled 309 patients aged 60 to 75 years with newly diagnosed t-AML or AML-MRC, two subtypes historically associated with poor prognosis and resistance to standard chemotherapy. Patients were randomized to receive either CPX-351, a liposomal encapsulation of cytarabine and daunorubicin at a synergistic 5:1 molar ratio, or conventional 7 + 3 induction therapy (cytarabine 100 mg/m^2^/day for 7 days plus daunorubicin 60 mg/m^2^/day for 3 days). The primary endpoint was OS, with secondary endpoints including CR, EFS, and rates of hematopoietic stem cell transplantation [19,41].

Results from this trial demonstrated a statistically significant improvement in median OS for patients treated with CPX-351 (9.56 months) compared to those receiving 7 + 3 (5.95 months). The CR rate was also higher in the CPX-351 arm (47.7% vs. 33.3%), and more patients were able to proceed to HSCT (34% vs. 25%), which is a critical consideration in curative-intent strategies. Importantly, CPX-351 was associated with a longer time to neutrophil and platelet recovery, yet early mortality (within 60 days) was lower, suggesting a favorable safety profile despite delayed hematologic recovery. These findings led to FDA and EMA approval of CPX-351 for adults with t-AML or AML-MRC, establishing it as a new standard of care in this population [41].

Upon a 5-year follow-up, the improved OS with CPX-351 compared to 7 + 3 was sustained, corroborating prior evidence that CPX-351 may facilitate long-term remission and improved OS in patients aged 60–75 years with newly diagnosed high-risk or secondary acute myeloid leukemia [21,41].

### 7.4. Real-World Cohort Validation

On the same note, a multicenter real-world study revealed that CPX-351 provides great clinical benefit in high-risk AML, particularly t-AML and AML-MRC. Among 168 patients treated across France and Italy, CPX-351 achieved a 60% overall response rate, with 65% of evaluable responders reaching measurable residual disease (MRD) negativity. MRD status emerged as the strongest independent predictor of OS: patients with MRD < 10^−3^ had a median OS of 20.4 months compared to 12.9 months in MRD-positive cases (*p*-value of 0.006). Notably, this survival advantage persisted in multivariate analysis (HR = 2.6; *p*-value of 0.013), highlighting the prognostic value of MRD. Among transplant recipients, MRD-negative patients showed a trend toward improved OS (not reached vs. 26 months; *p*-value of 0.06), although statistical significance was limited by sample size. The study also highlighted the predominance of adverse-risk genetics per ELN 2022 (84%) and frequent mutations in *TP53*, *RUNX1*, and *ASXL1*. Overall, these long-term data reinforce CPX-351’s role in inducing deep remissions and improving survival in real-world high-risk AML, and they support ongoing trials using MRD as a primary endpoint [28].

Additionally, real-world data from the CREST-UK study further substantiates the clinical utility of CPX-351 in newly diagnosed t-AML and AML-MRC, complementing prior phase 3 trial evidence. In a cohort of 147 UK patients treated outside of clinical trial settings, CPX-351 achieved a CR/CRi rate of 53%, with a median OS of 12.8 months (95% CI: 9.2–15.3). Notably, 34% of patients proceeded to hematopoietic cell transplantation (HCT), with landmarked OS from the date of HCT not reached, and 2-year survival exceeding 70%. Among patients who achieved CR/CRi without HCT, median OS was 14.0 months, highlighting the regimen’s efficacy independent of transplant. CPX-351 was well tolerated, with no new safety signals identified, and outpatient administration proved feasible, reducing hospitalization duration across all treatment phases. These findings reinforce CPX-351’s role as a viable first-line option in high-risk AML, offering durable remissions and logistical advantages in real-world practice [27].

### 7.5. Comparative Efficacy in Younger Adults

The UK NCRI AML19 trial randomized 189 younger adults (median age 56) with newly diagnosed adverse cytogenetic AML or high-risk MDS to receive either CPX-351 or FLAG-Ida. While FLAG-Ida achieved higher overall response rates after two induction cycles (77% vs. 64%), CPX-351 led to significantly longer relapse-free survival (22.1 vs. 8.35 months). OS (13.3 vs. 11.4 months) and EFS were not statistically different. Importantly, patients with myelodysplasia-related gene mutations (excluding *TP53*) experienced markedly improved OS with CPX-351 (38.4 vs. 16.3 months), suggesting a genomic subgroup that may benefit most from liposomal chemotherapy. CPX-351 also enabled more patients to proceed to allogeneic stem cell transplantation in first remission (64% vs. 48%), reinforcing its role in transplant-preparatory strategies [26].

Toxicity profiles revealed key differences with CPX-351 being associated with delayed platelet recovery after both induction cycles, particularly cycle 2, and longer hospitalization (median 35.5 vs. 27 days). On the other hand, FLAG-Ida required more blood product support and intravenous antibiotics, reflecting higher supportive care needs. Grade ≥ 3 nonhematologic toxicities were comparable between arms (18% vs. 21%). Neutrophil recovery was slower with CPX-351 in cycle 1 but faster in cycle 2, suggesting a more predictable recovery trajectory. These findings highlight that while FLAG-Ida may offer faster cytoreduction, CPX-351 provides more durable remissions with manageable toxicity, especially in genomically defined high-risk AML/MDS [26].

### 7.6. Post-Hoc Analyses Support CPX-351 as a Superior Induction and Consolidation Strategy in Older Adults with High-Risk AML

Furthermore, in a post hoc analysis in the Pivotal phase III trial evaluating older adults aged 60–75 with newly diagnosed high-risk or s-AML, CPX-351 demonstrated superior outcomes versus the conventional 7 + 3 chemotherapy among patients who achieved CR or CRi. CPX-351 was linked to significantly longer median OS in this subgroup (25.43 vs. 10.41 months; HR = 0.49; 95% CI, 0.31–0.77), with consistent benefit across AML subtypes (t-AML, AML-MRC), age groups, and prior hypomethylating agent exposure. Patients who achieved CR or CRi with CPX-351 had higher rates of hematopoietic cell transplantation and longer OS landmarked from transplant (not reached vs. 11.65 months; HR = 0.43; 95% CI, 0.21–0.89). Even among those who did not undergo transplantation, CPX-351 conferred a numerically longer OS (14.72 vs. 7.59 months; HR = 0.57; 95% CI, 0.31–1.03), suggesting that the pharmacologic properties of CPX-351 may contribute to deeper remissions and improved survival independent of transplant [25].

An additional, exploratory post hoc analysis was performed on the Phase III trial evaluating the consolidation outcomes in older adults (aged 60–75) with high-risk or s-AML who received CPX-351 versus standard cytarabine/daunorubicin regimens (7 + 3 induction followed by 5 + 2 consolidation). Among patients who achieved CR or CRi, those treated with CPX-351 through both induction and consolidation experienced significantly prolonged median OS (25.4 vs. 8.5 months; HR = 0.44, 95% CI: 0.25–0.77). This survival benefit was observed both in patients who proceeded to hematopoietic cell transplantation (HCT) and those who did not. Importantly, CPX-351 consolidation was well tolerated, with a safety profile consistent with prior findings and no deaths during consolidation. Although hematologic recovery was slower with CPX-351, adverse event rates were comparable to the 5 + 2 arm. Notably, over half of CPX-351 consolidation cycles were administered entirely in the outpatient setting, demonstrating feasibility without compromising outcomes. These findings support the role of CPX-351 not only in induction but also as an effective and manageable consolidation strategy in older patients with high-risk AML [24].

## 8. Therapeutic and Outcome-Based Comparison of CPX-351 Versus Conventional 7 + 3 Chemotherapy in AML

The comparative analysis of standard 7 + 3 chemotherapy and CPX-351 highlights the advancing treatment landscape in AML, especially for high-risk subtypes. CPX-351, a liposomal formulation of cytarabine and daunorubicin in a set 5:1 molar ratio, exhibits enhanced clinical outcomes in older persons with t-AML or acute myeloid leukemia with AML-MRC. In contrast to conventional induction regimens, CPX-351 demonstrates enhanced median and 5-year OS, increased rates of full remission and hematopoietic stem cell transplantation, along with diminished early mortality. Although hematologic recovery is longer, the safety profile indicates diminished cardiotoxicity and lower infection rates. These data endorse CPX-351 as a preferred frontline treatment for specific AML groups, consistent with precision-based therapeutic approaches [33,48,49].

The following summary table (Table 3) contrasts conventional cytarabine/daunorubicin chemotherapy with CPX-351 across formulation, dosing, survival outcomes, remission rates, safety, and applicability to special populations. CPX-351 demonstrates improved survival, higher remission and transplant rates, and reduced early mortality, particularly in older adults with t-AML or AML-MRC.

## 9. Limitations and Future Perspectives

Limitations of CPX-351 include its restricted indication to newly diagnosed t-AML and AML-MRC, primarily in older adults (ages 60–75), as established by pivotal phase 3 trials [21,41]. Its benefit in other AML subtypes, younger patients, or in relapsed/refractory settings remains less well defined. CPX-351 is associated with prolonged myelosuppression, leading to delayed neutrophil and platelet recovery, which may increase infection risk and require extended supportive care [21,41]. Although nonhematologic adverse events are comparable to conventional chemotherapy, early mortality, and cardiac toxicity, especially at higher doses in elderly patients, remain concerns [41,50]. Additionally, outcomes for patients with adverse-risk cytogenetics or molecular features, such as *TP53* mutations, are still suboptimal despite CPX-351’s improved efficacy over 7 + 3 [49,51].

Future research directions focus on expanding CPX-351’s use beyond its current indications. Ongoing studies are evaluating its role in higher-risk myelodysplastic syndromes and chronic myelomonocytic leukemia, both upfront and after hypomethylating agent failure, as well as optimal dosing strategies for frail or elderly patients [50,52]. Combination regimens with targeted agents, such as *FLT3* inhibitors and *CHK1* inhibitors, are under investigation to enhance efficacy, particularly in molecularly defined subgroups [51,53]. Randomized trials comparing CPX-351 with hypo-methylating agents, targeted therapies, and allogeneic stem cell transplantation are warranted to clarify its place in evolving AML treatment algorithms [7,52]. Further research is needed to elucidate mechanisms of resistance and to identify biomarkers predictive of response [7,39].

## 10. Conclusions

CPX-351 signifies notable improvements in the OS and remission over traditional 7 + 3 chemotherapy for managing high-risk or secondary AML, especially in elderly patients. The liposomal combination of cytarabine and daunorubicin in CPX-351 preserves a synergistic 5:1 molar ratio, leading to enhanced pharmacokinetics and targeted bone marrow delivery, which results in increased full remission rates and prolonged overall life relative to the conventional method. CPX-351 is characterized by a reduced early mortality rate and a similar safety profile, but with a delayed hematologic recovery, and has shown sustained survival advantages at five years [7,21,39,41,54].

The clinical advantage of CPX-351 is mainly evident in patients with t-AML or AML with myelodysplasia-related alterations, where traditional chemotherapy has typically produced suboptimal results. It enhances the likelihood of successful hematopoietic stem cell transplantation, resulting in better post-transplant survival rates. These results have resulted in its endorsement as the optimal induction regimen for high-risk or secondary AML in adults, which is corroborated by randomized phase 3 studies and comprehensive follow-up analysis [16,20].

## Figures and Tables

**Table 1:** Summary of CPX-351 clinical trials and real-world studies across diverse AML populations.

Study	Population	Setting	Dosing	Efficacy	Survival Outcomes	Safety Profile	Notable Insights
Feldman et al., 2011 (Phase I) [18]	48 patients (43 AML, relapsed/refractory)	First-in-human dose escalation	Days 1, 3, 5; MTD = 101 u/m^2^	23% CR/CRp in AML; responses at ≥32 u/m^2^	Median remission: 6.9 mo	DLTs: CHF, HTN crisis, prolonged cytopenias; rash in 71%	5:1 ratio maintained ≥24 h; prolonged half-lives (Ara-C ~31 h, DNR ~22 h); active in heavily pretreated AML
Lancet et al., 2014 (Phase II) [19]	126 older adults (60–75 years) with untreated AML	CPX-351 vs. 7 + 3	CPX-351: 100 u/m^2^ (D1,3,5); consolidation allowed	CR/CRi: 66.7% vs. 51.2%; sAML CR/CRi: 57.6% vs. 31.6%	OS: 14.7 vs. 12.99 mo; sAML OS: 12.1 vs. 6.19 mo (HR 0.46)	Slower ANC/platelet recovery; more grade 3–4 infections but lower 60-day mortality	CPX-351 reduced 60-day mortality (4.7% vs. 14.6%); improved OS and EFS in sAML; supports phase III development
Cortes et al., 2015 (Phase II) [20]	125 adults (18–65 years) with first-relapse AML	CPX-351 vs. investigator’s choice salvage	CPX-351: 100 u/m^2^ (D1,3,5); consolidation allowed	CR/CRi: 49.4% vs. 40.9%; CR: 37% vs. 31.8%	OS: 8.5 vs. 6.3 mo; 1-year OS: 36% vs. 27%; poor-risk OS: 6.6 vs. 4.2 mo (HR 0.55, *p* = 0.02)	Similar early mortality; slower ANC/platelet recovery; infection-related AEs common	CPX-351 improved OS and EFS in poor-risk patients; liposomal delivery may bypass resistance mechanisms
Lancet et al., 2021 (Phase III) [21]	309 older adults (60–75 years) with newly diagnosed sAML	CPX-351 vs. 7 + 3 induction	CPX-351: 100 u/m^2^ (D1,3,5); consolidation 65 u/m^2^ (D1,3)	CR/CRi: 47.7% vs. 33.3% (7 + 3); CR: 37.3% vs. 25.6%	OS: 9.56 vs. 5.95 mo (HR 0.69, *p* = 0.003); EFS: 2.53 vs. 1.31 mo	Comparable AE rates; longer cytopenias with CPX-351	OS benefit across age/subtypes; higher HCT rate (34% vs. 25%); supports CPX-351 as new standard in sAML
Krauss et al., 2019 (FDA Approval) [22]	309 patients (60–75 years) with t-AML or AML-MRC	Regulatory review of Study 301	CPX-351: 100 u/m^2^ (D1,3,5); consolidation 65 u/m^2^ (D1,3)	CR: 38% vs. 26% (7 + 3)	OS: 9.6 vs. 5.9 mo (HR 0.69, *p* = 0.005); OS in t-AML: HR 0.48; AML-MRC: HR 0.70	Similar AE profile to 7 + 3; more prolonged cytopenias and bleeding risk	First FDA-approved therapy for t-AML/AML-MRC; boxed warnings for copper load, cytopenias, and formulation interchangeability
Issa et al., 2020 (Phase II) [23]	56 high-risk AML patients (median age 69)	Newly diagnosed AML with high induction mortality risk	50, 75, 100 u/m^2^ on days 1, 3, 5	CR/CRi: 19% (50), 38% (75), 44% (100)	OS: 4.3 (50), 8.6 (75), 6.2 mo (100)	TEAEs: FN (34%), pneumonia (23%), sepsis (16%)	75 u/m^2^ had best efficacy/safety balance; *TP53* mutation = poor OS (2.6 mo); supports use in frail patients
Kolitz et al., 2020 (Consolidation) [24]	81 patients with CR/CRi post-induction	CPX-351 vs. 7 + 3/5 + 2 consolidation	CPX-351: 65 u/m^2^ (D1,3); 5 + 2: cytarabine + daunorubicin	OS: 25.4 vs. 8.5 mo (HR 0.44); OS post-HCT: NR vs. 9.8 mo; OS without HCT: 13.7 vs. 8.4 mo	No deaths during consolidation; relapse rates lower with CPX-351	TEAEs: FN (29%), pneumonia (8%), cellulitis (8%); slower ANC/platelet recovery	CPX-351 consolidation extended OS; outpatient delivery feasible in 51–61%; supports full-cycle CPX-351 strategy
Lin et al., 2021 (Post Hoc) [25]	Subgroup of 125 patients who achieved CR/CRi in Lancet trial	Exploratory analysis of remission impact	Same as Lancet (induction + consolidation)	CR/CRi: 48% (CPX-351) vs. 33% (7 + 3); CR: 37% vs. 26%	OS: 25.4 vs. 10.4 mo (HR 0.49); OS in t-AML: not reached vs. 9.15 mo (HR 0.21); OS in AML-MRC: 19.2 vs. 11.0 mo	Safety profile consistent with 7 + 3; longer cytopenias	CPX-351 led to deeper remissions and longer OS across subgroups, even without HCT; supports durable benefit in responders
Othman et al., 2023 (AML19 Trial) [26]	189 younger adults (<60 years) with adverse karyotype AML or high-risk MDS	CPX-351 vs. FLAG-Ida	CPX-351: 2 induction + 2 consolidation cycles; FLAG-Ida: fludarabine, cytarabine, idarubicin	CR/CRi: 64% vs. 76%; OS: 13.3 vs. 11.4 mo (NS); RFS: 22.1 vs. 8.35 mo (HR 0.58, *p* = 0.03)	OS not significantly different; RFS improved with CPX-351; more SCT in CR with CPX-351	Comparable grade ≥ 3 toxicity; longer platelet recovery with CPX-351; less death in remission	CPX-351 improved RFS and OS in patients with MDS-related gene mutations (median OS: 38.4 vs. 16.3 mo); supports genomic stratification
Mehta et al., 2024 (CREST-UK) [27]	147 UK patients with newly diagnosed t-AML or AML-MRC (38% < 60 years)	Real-world, multicenter, outpatient-capable	CPX-351: 100 u/m^2^ (induction), 65 u/m^2^ (consolidation)	CR/CRi: 53%; MRD negativity in 51% of tested responders	OS: 12.8 mo overall; 25.4 mo post-HCT; 14.0 mo in CR/CRi without HCT; OS in *TP53*-mutated: 4.5 mo	Grade ≥ 3 TEAEs in 75%; FN (38%), pneumonia (8%), sepsis (7%); cardiac TEAEs in 12%	Outpatient delivery feasible; reduced hospitalization days; OS benefit preserved across age and risk strata; *TP53* mutation = poor prognosis
Cluzeau et al., 2025 (RWE) [28]	168 adults with newly diagnosed t-AML or AML-MRC	Real-world, multicenter, long-term follow-up	CPX-351: 1–2 induction cycles; consolidation ± HSCT	CR/CRi: 60%; MRD negativity in 65% of tested responders	OS: 13.3 mo overall; OS with MRD < 10^−3^: 20.4 mo vs. 12.9 mo (*p* = 0.006); OS post-HCT: NR vs. 26 mo	Acceptable safety; MRD > 10^−3^ independently predicted poorer OS (HR 2.6)	MRD negativity strongly associated with improved OS

Abbreviations: AML, acute myeloid leukemia; sAML, secondary AML; t-AML, therapy-related AML; AML-MRC, AML with myelodysplasia-related changes; MDS, myelodysplastic syndrome; CMML, chronic myelomonocytic leukemia; CR, complete remission; CRi, complete remission with incomplete hematologic recovery; CRp, complete remission with incomplete platelet recovery; ORR, overall response rate; MRD, measurable residual disease; HSCT/HCT, hematopoietic stem cell transplantation; OS, overall survival; EFS, event-free survival; RFS, relapse-free survival; HR, hazard ratio; TEAE, treatment-emergent adverse event; FN, febrile neutropenia; ANC, absolute neutrophil count; WBC, white blood cell; RIC, reduced-intensity conditioning; MAC, myeloablative conditioning; NGS, next-generation sequencing; IWG, International Working Group; ELN, European Leukemia Net; WHO, World Health Organization; ICC, International Consensus Classification; LAIP, leukemia-associated immunophenotype; MFC, multiparameter flow cytometry; PCR, polymerase chain reaction; CTCAE, Common Terminology Criteria for Adverse Events; NR, not reached; u/m^2^, units per square meter; D1,3,5, days 1, 3, and 5 of treatment; FLAG-Ida, fludarabine, cytarabine, G-CSF, and idarubicin regimen; 5 + 2, cytarabine + daunorubicin consolidation regimen; Lindsley classifier, genomic classifier for AML subtypes; pan-AML, de novo AML without secondary-type mutations or *TP53* mutation [18,19,20,21,22,23,24,25,26,27,28].

**Table 2:** Comparison of Conventional Cytarabine (7 + 3) Versus CPX-351 (Vyxeos) in Induction and Consolidation Regimens for AML Treatment [21,41,44,45,46].

Parameter	Conventional Cytarabine (7 + 3)	CPX-351 (Vyxeos)
Induction Dose	100 mg/m^2^/day	100 units/m^2^ (cytarabine 100 mg/m^2^ + daunorubicin 44 mg/m^2^)
Induction Schedule	Daily for 7 consecutive days	Days 1, 3, and 5 (90 min infusion)
Route of Administration	Continuous IV infusion (standard); also, IV bolus, SC, or IT in specific contexts	IV infusion over 90 min
Consolidation Dose	Variable; may include high-dose cytarabine (1000–3000 mg/m^2^ per dose)	65 units/m^2^ (cytarabine 65 mg/m^2^ + daunorubicin 29 mg/m^2^)
Consolidation Schedule	Depends on protocol; often multiple cycles	Days 1 and 3 (90 min infusion)
Intrathecal Use	Occasionally used for CNS prophylaxis or treatment	Not used intrathecally
Formulation	Free cytarabine	Liposomal encapsulation of fixed 5:1 molar ratio of cytarabine and daunorubicin

**Table 3:** Comparison of conventional cytarabine (7 + 3) and CPX-351 (Vyxeos) across formulation, dosing, survival outcomes, remission rates, safety, and applicability to special populations [33,48,49].

Features	Conventional Chemotherapy (7 + 3)	CPX-351 (Liposomal Cytarabine/Daunorubicin)
Drug Formulation	Free cytarabine + daunorubicin	Liposomal cytarabine + daunorubicin (5:1)
Indication	All AML subtypes, especially de novo AML	High-risk/secondary AML (AML-MRC, t-AML)
Dosing (Induction)	Cytarabine 100 mg/m^2^/day × 7 days (CI) + daunorubicin 60 mg/m^2^/day × 3 days (IV)	100 units/m^2^ IV over 90 min on days 1, 3, 5 (cycle 1); days 1, 3 (cycle 2)
Dosing (Consolidation)	5 + 2 regimen or intermediate/high-dose cytarabine	65 units/m^2^ IV over 90 min on days 1, 3
Median OS	5.95–6.2 months	9.33–10.3 months
5-Year OS	8%	18%
Complete Remission Rate (CR/CRi)	33–39%	47–49%
HSCT Rate (Post-Remission)	Lower	Higher
Early Mortality (30-day)	10.6%	4.9–5.9%
Safety Profile	Myelosuppression, infection, cardiotoxicity	Similar myelosuppression, less cardiotoxicity, fewer infections
Recovery of Counts	Faster	Slower
Special Populations	All AML, including younger adults	Older adults (60–75) with AML-MRC/t-AML

## Data Availability

No new data were created or analyzed in this study.
